# A Parallel Modular Biomimetic Cilia Sorting Platform

**DOI:** 10.3390/biomimetics3020005

**Published:** 2018-03-30

**Authors:** James G. H. Whiting, Richard Mayne, Andrew Adamatzky

**Affiliations:** Unconventional Computing Laboratory, University of the West of England, Bristol BS16 1QY, UK; Richard.Mayne@uwe.ac.uk (R.M.); Andrew.Adamatzky@uwe.ac.uk (A.A.)

**Keywords:** cilia, parallel actuator, *Paramecia*, sorting

## Abstract

The aquatic unicellular organism *Paramecium caudatum* uses cilia to swim around its environment and to graze on food particles and bacteria. *Paramecia* use waves of ciliary beating for locomotion, intake of food particles and sensing. There is some evidence that *Paramecia* pre-sort food particles by discarding larger particles, but intake the particles matching their mouth cavity. Most prior attempts to mimic cilia-based manipulation merely mimicked the overall action rather than the beating of cilia. The majority of massive-parallel actuators are controlled by a central computer; however, a distributed control would be far more true-to-life. We propose and test a distributed parallel cilia platform where each actuating unit is autonomous, yet exchanging information with its closest neighboring units. The units are arranged in a hexagonal array. Each unit is a tileable circuit board, with a microprocessor, color-based object sensor and servo-actuated biomimetic cilia actuator. Localized synchronous communication between cilia allowed for the emergence of coordinated action, moving different colored objects together. The coordinated beating action was capable of moving objects up to 4 cm/s at its highest beating frequency; however, objects were moved at a speed proportional to the beat frequency. Using the local communication, we were able to detect the shape of objects and rotating an object using edge detection was performed; however, lateral manipulation using shape information was unsuccessful.

## 1. Introduction

Motile cilia are finger-like projections of a cell found on a variety of eukaryotic organisms, including unicellular protists (the `ciliates’) and animals. The purpose of these organelles, which are up to 10 μm long and less than 1 μm in width, is to generate fluid currents in surrounding media. In ciliates, this serves to propel the organism as a means of motility, enhance feeding through concentrating and `sorting’ adjacent particles [1,2,3,4] and enable environmental sensing [5]. In humans, they are present in epithelial cells such as those found lining most of the respiratory surfaces (to drive the mucociliary escalator) and the fallopian tube (to aid ova transport).

**Figure 1:**
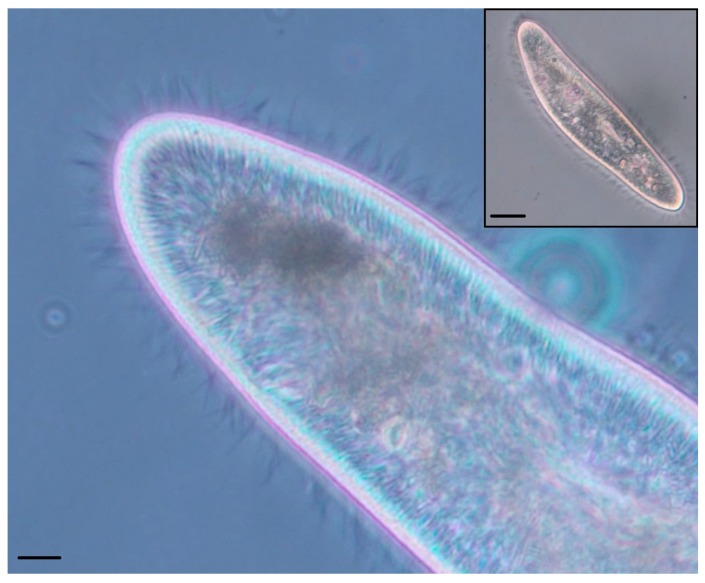
Phase contrast photomicrographs of a *Paramecium caudatum*. (Main) Anterior tip of the organism, showing hundreds of cilia (hair-like appendages) sprouting from its surface. Scale bar: 10 μm. (Inset) Lower magnification view showing the whole organism. Scale bar: 25 μm.

Academic interest in cilia is justified through their incompletely understood emergent properties (sorting, establishment of asynchronous, or `metachronal’, waves) and their involvement in a range of acquired and inherited human pathologies (ciliopathies). In this investigation, we take inspiration from the cilia of *Paramecium caudatum* (Figure 1) and fabricate a biomimetic sorting platform.

Cilia have been the topic of object manipulation by several research groups; while cilia on microorganisms are responsible for motility by creating vortexes in the surrounding fluid, thus propelling the organism around its environment, some ciliary sorting or at least particle manipulation does occur in the mucociliary escalator in human bronchial epithelial tissues to maintain a clear airway [6]. Several academic articles have been published that present microactuator arrays and micro-electrical-mechanical system (MEMS)-based sorting actuation [7], purportedly facilitating the manipulation of small objects such as surface mount electronic components. Alternative to MEMS technology is soft cilia, which mimic the whipping-like power and recovery stroke, albeit several orders of magnitude larger; these soft robotic systems use electroactive polymers that bend and move in a similar manner to biological cilia [8]. The structure of the soft robotic cilia is such that it is flexible enough to create the ciliary beat while too flexible to manipulate objects in air; these soft robotics, at least in their current form, are better suited to creating vortexes in liquid, and thus, the applications differ. With either soft robotics or MEMS cilia, the control of the system is derived from either pre-programed contraction timing or computer–camera feedback, respectively; this again limits the scalability as the computer–camera feedback needs to be recalibrated to encompass new cilia. We have previously published work using vibrating motors as actuators; however, while the distributed nature was advantageous, the biomimicry element was minimal. Although progress has been made towards motor control through the use of cellular automata-inspired control schemes, this has only been accomplished in swimming robots [9].

Distributed intelligence in sorting platforms and indeed other multinodal systems allows for the removal of a central control unit; emergent properties arising from a simple set of behavioral rules combined with distributed control can create apparent intelligence in very simple systems such as swarms or single-celled organisms [10]. It is the purpose of this work to create a biologically inspired cilia, which mimics the biological ciliary structure, assembly and beating characteristics from a specific organism of interest (*P. caudatum*) while creating a plug-and-play platform with distributed and emergent object manipulation.

Autonomous sorting platforms are used in a wide array of applications; from the food industry to mineral mining or even in distribution systems. The large majority of these platforms are either open-loop or function with minimal sensor feedback. The lack of feedback can be advantageous to reduce both running and resource cost; however, this means systems are designed solely for a specific task such as with electro-mechanical vibratory bowl feeders [7,11,12,13]. Open-loop sorting technology performs well at simple tasks; however, if the parameters change, then the entire system needs to be redesigned or at least recalibrated. The ability to repurpose a sorting platform on-the-fly or even program in several different operating modes is a key benefit of an adaptable system; closed-loop systems can provide such adaptability. Closed-loop systems may also reduce errors and allow sorting of objects with physically similar dimensions that would otherwise not be sorted. Modern conveyor belt closed-loop systems employ cameras and other sensing devices that facilitate the sorting of objects. The mail sorting room of the British Royal Mail uses an automated sorting system called Integrated Mail Processing (IMP) and is, as the name suggests, an integrated system of traditional vibratory bowl feeders, conveyor distribution systems and high-resolution camera systems all controlled by banks of computers. This system is very efficient, sorting a letter in under 21 s when the correct address and post code is used; however, illegible handwriting and missing parts of the address means the erroneous mail has to be sorted by hand. This system, used as an example, is employed across the United Kingdom in sorting offices; however, it is designed for a very specific task; while being modular in design, it is far from adaptable, meaning the whole system is only suitable for sorting letters. We aim to develop a modular and easily reprogramable sorting platform that can complete a wide range of sorting tasks.

## 2. Materials and Methods

### 2.1. Design

Unless otherwise stated, we refer to Wichterman’s opus on the biology of *Paramecium* in this section; see [14]. *P. caudatum* is a freshwater microorganism measuring approximately 250 × 50 μm, typically coated in 5000–6000 cilia. The purpose of these cilia is primarily to generate motile force, but also, they play a role in drawing food towards the oral cavity of the organism. In laboratory experiments, it has been demonstrated that various modes of ciliary beating exist to enhance foraging behavior and may be classified as differential manipulation of proximate particulate matter (i.e., sorting) [1]. To expand, when not actively migrating, ciliary beating patterns alter in order to simultaneously draw small ingestible-sized particles towards its mouth, eject uningested particles in fluid contrails and hold larger particles static in close proximity (likely by the formation of an electrostatic double layer) in order to strip smaller particulates from them.

**Figure 2:**
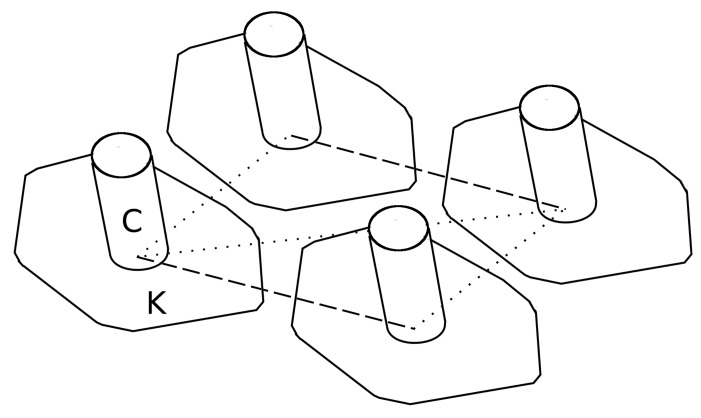
Schematic diagram to demonstrate the location within kinetid basins, from the top-down view of the *P. caudatum* pellicle. C: Cilium; K: Kinetid; dashed line: Kinetodesmal fibrils; dotted line: infraciliature.

*P. caudatum* cilia protrude through the outer surface of the organism, which is coated by a skin-like layer called the pellicle, while maintaining continuity with its external membrane. The pellicle has properties akin to a deformable, semi-permeable exoskeleton, which delineates clear basins through which the cilia protrude; these basins, which also house other organelles, are tiled hexagonally and are demarcated internally by protein structures (microtubule bundles in ectoplasmic ridges) to form structures known as kinetids (Figure 2). Internal ciliary structures anchor deep within the gel-like layer that sits below the pellicle (ectoplasm) via a basal body, and further protein structures (microtubules and actin microfilaments, collectively known as infraciliature) link rows of cilia together in extremely complex, intricate patterns. It is still unknown exactly how the coupling between cilia in the organism’s dense fibrillar networks affects beating characteristics, although it has been demonstrated that movements in the class of large multiprotein fibres (kinetodesmal fibrils) that link cilia together in rows are coupled to contractile hardware, which provides some of the motive force for beating activity.

**Figure 3:**
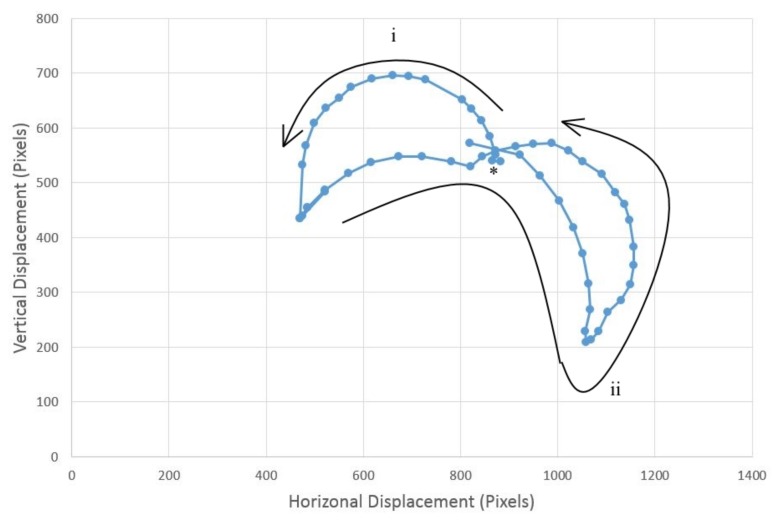
The full ciliary beat action performed by the artificial cilium. The power stroke (i) starts at the marked position (*), which progresses into the recovery stroke (ii).

Cilia beat with a rhythmic whip-like motion (Figure 3) of beating that comprises a rigid power stroke, which drives the fluid current, and a passive recovery stroke, where the cilium returns to its original position via bending in all dimensions in a cardioid pattern. The aforementioned phenomenon of asynchronous (metachronal) ciliary beating in wave-like patterns has long since been studied, but no concrete mechanisms behind their instigation or coordination have yet been widely accepted; contemporary evidence on the issue, based on computer modeling, emphasizes the importance of local hydrodynamic interactions between neighboring cilia on the process [15] (as each cilium is capable of multimodal sensing), but internal physical linkage is also thought to play a role.

The artificial cilia presented in this investigation were based on the following principles in order to mimic the biological equivalent as closely as possible, hence also demonstrating the novelty of our approach:

1. Hexagonal tiling, to mimic the kinetid structure
2. Three-dimensional bending and about-axis rotation
3. Local neighbor communication
4. Discrete sensing for each cilium

### 2.2. Method

**Figure 4:**
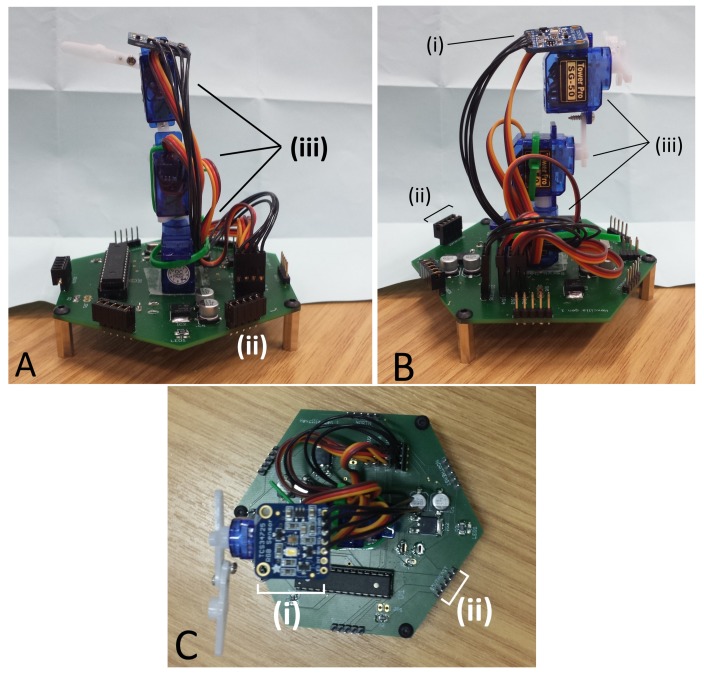
Three views (A,B and C) of an artificial cilia developed to mimic ciliary beating. (i) The color sensor; (ii) the local neighbor pins; (iii) servos mounted in line.

To create biomimetic cilia movement, we produced a hinged servo robotic arm, consisting of three servos. Three Tower Pro SG50 Micro Digital Servos (Servo Shop, Frodsham, UK) were used to copy the whipping power and recovery stroke, with two servos mounted axially to create a two-segment robotic arm, and the third servo placed orthogonally at the base of the arm, to facilitate rotation of the cilia to stroke in different directions; this setup is shown in Figure 4. The servos are connected to each other with the manufacturer-provided servo arms and screws. Each servo is controlled by an Atmega 328 8-bit AVR RISC-based microcontroller (RS Components, Corby, UK), with pulse-width-modulation angular control for each servo. The ciliary beating action was created by a sequence of angular controls to each servo in the armature in order to create the typical asymmetrical ciliary beating action. Each ciliary unit in the form of servo armature and relative microcontroller is mounted on a printed circuit board. The overall force of the three-servo cilia was recorded 12 times using a Sauter FK50 force gauge (RS Components, Corby, UK) attached to the tip of the cilia during the power stroke phase. All the files required to reproduce and reprogram the cilia can be obtained and are provided as open-source material for anyone to use provided citations and credit are given where appropriate (https://github.com/UnconventionalComputingUWE/Paramecium).

#### 2.2.1. Assembly

**Figure 5:**
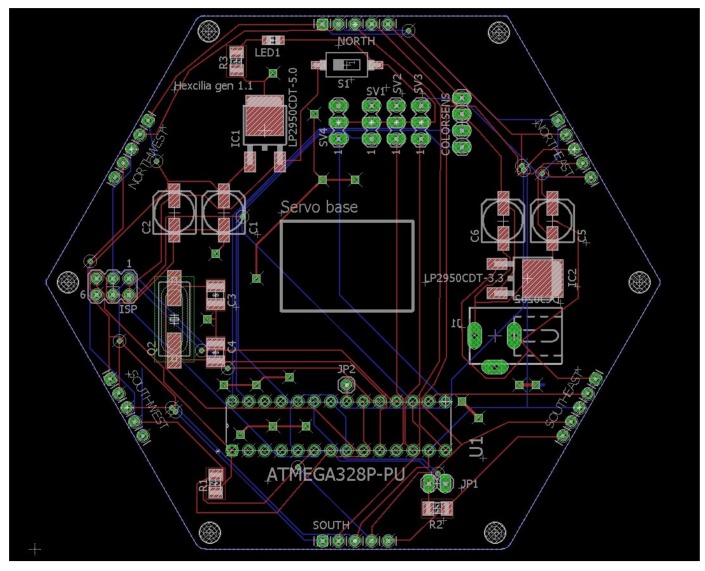
Hexagonal cilia board layout for the Printed Circuit Board created using EagleCAD design software (Autodesk, San Rafael, CA, USA).

The circuit board is a mixture of surface mount devices (SMD) and through-hole components (Figure 5); while surface mount components are often assembled by a pick-and-place machine, the components used in this project are easily assembled manually by hand by someone with reasonable soldering experience, as larger components have been purposefully chosen. Servos were mounted on to the board using super glue gel, in order to hold them in place during manipulation of heavier objects. Servo armatures were attached to the servo above using cable ties or super glue gel, and both provided acceptable strength of attachment. The ciliary tip was an extra long servo armature assembled by connecting two linkages together using screws provided with the servos. The color sensor board was again attached to the top using super glue gel. Each servo and the color sensor board were connected to the board using pin headers to facilitate simple swapping of servos.

The organism’s membrane holds the cilia in alignment in the continuous outer membrane; under sufficient magnification, a hexagonal tessellation of longitudinal rows is visible (Figure 2), with one cilia protruding from each hexagonal structure. It is because of this hexagonal tessellation that each ciliary unit’s circuit board has hexagonal outer dimensions. This tessellation provides opportunities for sharing power, providing a system that is entirely reconfigurable in morphology; additionally, it is significantly biomimetic in geometry [14]. Each Printed Circuit Board (PCB) measured 95 × 83 mm, and a total of 35 boards was used to cover an area of approximately 50 × 39 cm. Due to the power sharing design of the boards, the number of boards that can be supported is limited to the sole power supply, rather than the number of individual power supplies to each board. In order to share data with its six neighboring cells, the boards have five pin headers along each edge of the board, and these align with pins on adjacent boards. Five pin jumper modules connect to digital Input/Output (I/O) pins on the microprocessor, which facilitates neighbor communication. Recording of the objects’ position and speed was performed in real time using a webcam mounted on a clamp stand above the assembled platform, with video being fed to a custom MATLAB (Mathworks, Natick, MA, USA) script for tracking object position. In order to facilitate smooth movement, the cilia shared data to coordinate beating. The Matlab script can be supplied by the Corresponding Author.

#### 2.2.2. Color Detection

*Paramecium* grazes on food particles of different sizes and actively hunts for bacteria and minute protozoa, funneling them into the oral grove where they are sorted into edible and inedible and are passed into food vacuoles via the buccal cavity or rejected, respectively. As the focus of this work is to create a bioinspired sorting platform, a variety of sensors could be used, dependant on the practical application, of which there is a huge variety; the microcontroller can interact with a huge array of analogue or digital sensors, so desired sensing systems can be easily integrated for each application. In this instance, however, color sensors were employed to differentiate between objects. For simplicity, an Adafruit TCS34725 RGB color sensor (Adafruit, New York, NY, USA) was employed. The sensor has an inbuilt light-emitting diode (LED) and infrared (IR) filter to accurately detect a 3,800,000:1 dynamic range of color. The sensor board and supporting electronics were mounted atop the distal servo for optimum color detection, but did not extend above the servo’s working armature.

#### 2.2.3. Software

The embedded code on the microprocessor controls the system’s behavior; like the emergent intelligence of the *Paramecia*, a simple set of rules governs the organism’s response, leading to an apparently complex behavior. The software reads the value of the RGB sensor, and if a colored object is detected, the cilia will act; the base servo will rotate to an angle based on the relative RGB values and then perform a power and recovery stroke in that direction; this action is repeated until the RGB sensor no longer detects a colored object. Each cilia acts alone, but together, they are capable of transporting objects across the entire platform as each element knows its own behavior. It is possible, again if the application would desire it, to program each cilia or sections of cilia to act in different ways so as to sort objects in more complex manners, or with different sensors, to sort color and then weight, for example. In this instance, if the RGB sensor detects a red object, the cilia beats in a north-easterly direction (relative to the board); if a green object is identified, the cilia beats in a north-westerly direction; and if a blue object is detected, then the cilia beats in a south-westerly direction. It is possible to beat in any direction given a range of color values; however, for this proof-of-concept design, only three colors were used.

Investigating the beat frequency and the speed of locomotion of sorted objects, we tested objects moving at four different ciliary beat frequencies, those with periods of 0.7, 1.2, 1.6, 2.4 and 5.3 s. The object that was moved was a 20 × 15 cm jiffy bag with small electronic components inside, something typical of a mail sorting room situation; three different colors of jiffy bags were used to enable sorting in different directions, and all weighed the same (127 g) to eliminate bias between parcels. Each color parcel sorting was repeated five times, and the parcel was placed in the middle of the ciliary surface for each repeat.

In addition to sharing power, each circuit board has a connection to each of its neighbors. Much like cellular automaton, which uses neighbor states to sequentially evolve behavior, the states of each neighbor cilia can be detected. This feature facilitates the ability to produce the familiar ciliary metachronal wave. The nature of cilia-to-cilia connections is incompletely elucidated, although they are physically linked by parallel fibrils running through the cell cortex, kinetodesma, chemically linked propagating ionotropic signals and also hydrodynamically coupled to each other via the fluid movements they induce [16,17]. We mimicked these forms of connectivity with local neighbor connections. Each cilia Printed Circuit Board (PCB) has an output, to announce its state to all six neighbors, and six inputs with which it detects each of its neighbor states. We have programed the embedded software in such a way to reproduce the metachronal waves; using the detection of an active neighbor cilia to initiate a phase lagged power stroke. This lagged ciliary beating action creates the metachronal wave.

**Figure 6:**
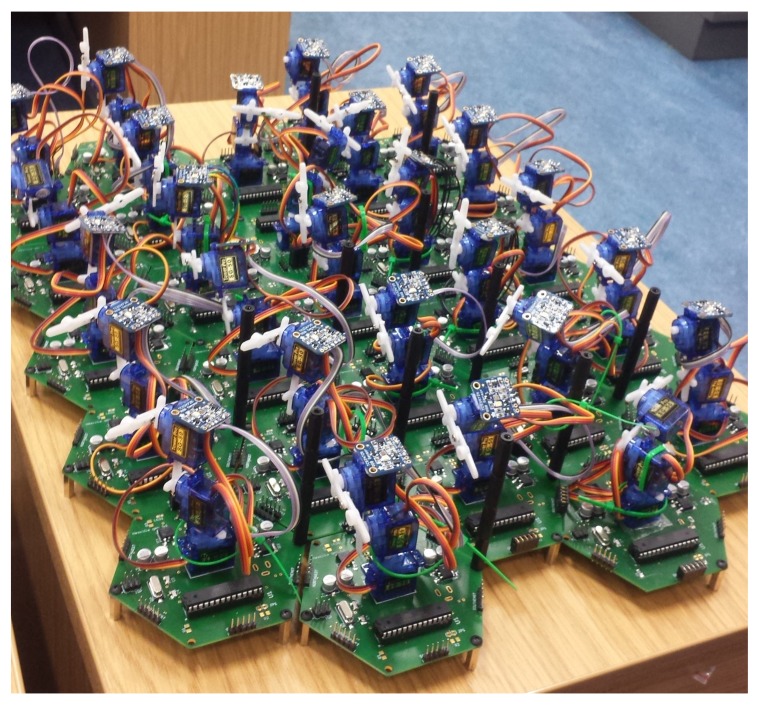
Multiple-tiled hexagonal cilia connected to each neighbour, sharing power and data.

In order to detect and sort based on object shape, the sorting platform must be able to identify different shapes. This can be most accurately performed with edge detection. As each cilia cell is able to communicate and share information with its local neighbor, it is able to determine which of its surrounding cells are “occupied”. Using the ability to detect colored objects above its own headspace, each cell knows if an object is above it, then by sharing this information with its neighbor as shown in Figure 6, it is able to determine if it is part of an edge of an object or part of an inner cell. Cells with at least one or more neighbors that are not “occupied” form part of the edge. If all cells in the platform perform this check, then the surface is able to identify the outline of an object.

**Figure 7:**
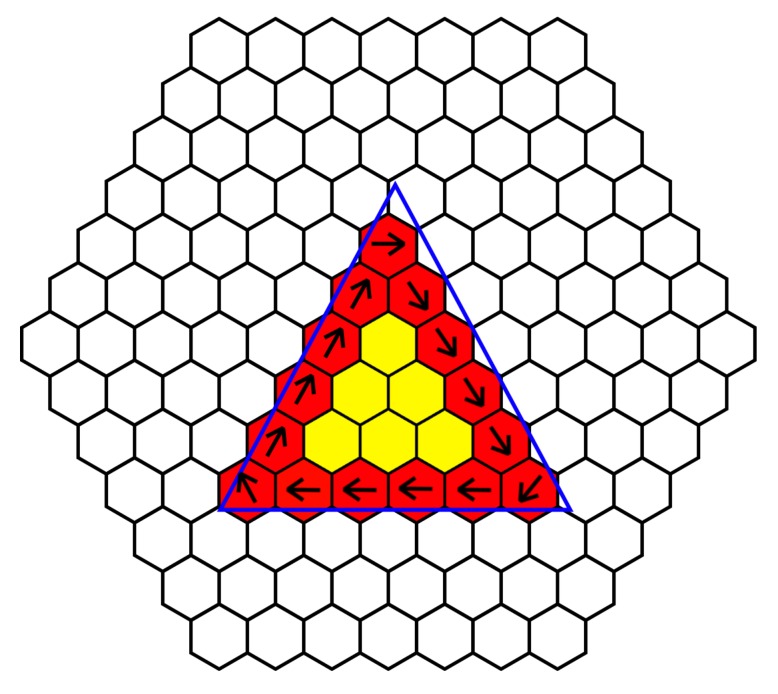
Rotation utilizing edge detection, where neighbors share information to detect the edge of an object and perform perpendicular beating to rotate an object. Red cells indicate edges, while yellow cells indicate an internal cell. Black arrows indicate the direction of ciliary beating and the blue triangle is where the object is placed, to illustrate placement of object.

Rotation of an object is another task that can be performed by this platform. In order to perform rotation of an object about its origin, computation must be performed in order to determine either the outer edge or the center mass. Since we have neighbor communication and the center mass of a larger object could be buried several cells from the edge, we must determine the edge of an object. As we have discussed, we are able to determine the edge of a shape, by using the “occupied” status of each cell and its local neighbor, and we then take this one step further by programing in ciliary behavior when the cell is part of an edge. To rotate an object, all edge cell’s ciliary action must be coordinated; an edge cell was programed to beat the ciliary arm in a direction perpendicular to the edge and at clockwise orientation to the edge. Edge angle is calculated by averaging the local “occupied” neighbors. Figure 7 demonstrates the process, giving several examples of this behavior. Cells in the center of the object do not respond, as the edge cells’ ciliary beating is sufficient to rotate the object.

#### 2.2.4. Shape Detection

We attempted to identify an object’s shape using local communication and neighbors’ states. While we can detect the edge of an object, determining the shape provided much more of a challenge. Since each cell knows what state its neighbor is in and, importantly, what direction each neighbor is in, we can determine the number of active cells around each cell; using this information, we created a heat map of cells with the color proportional to the number of active neighbor cells. However, there was a limitation of sharing the neighbor states with anything but the local neighbors; increasing the local communication from each neighbor to include the neighboring cell’s neighbors increases the number of cells from 6 to 18, a three-fold increase in communication and geometric computational complexity. In another attempt to determine the shape, we developed a method to identify an object’s corner and, using local communication, share the number of corners in a local area. Corners or at least significant changes in shape can be identified using the number and position of neighboring active cells.

## 3. Results

**Figure 8:**
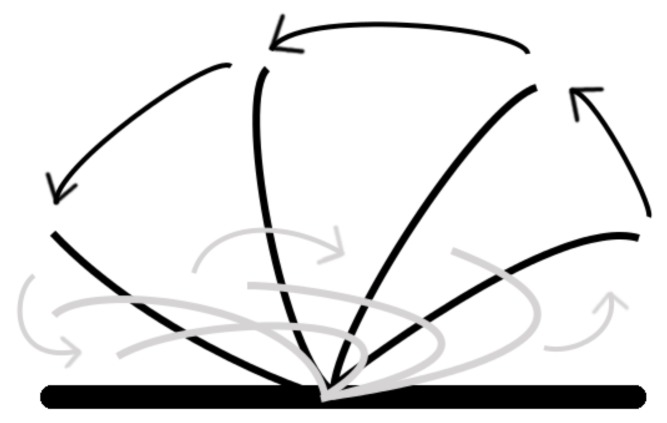
A typical ciliary beat cycle showing the power stroke in black and the recovery stroke in grey.

**Figure 9:**
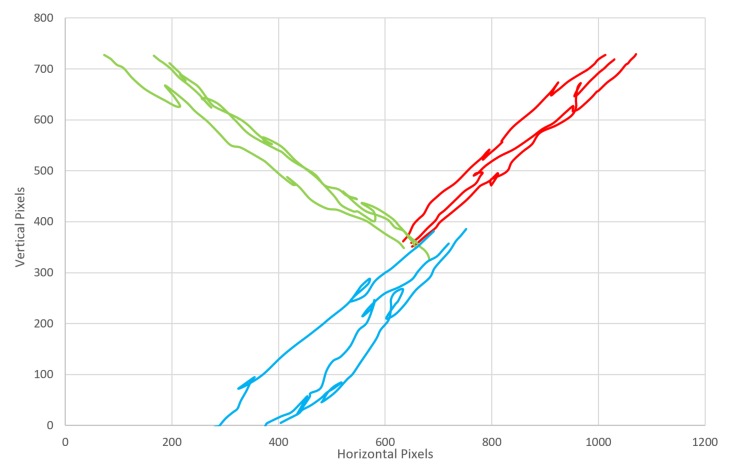
The direction of object movement. Three examples of red, green, and blue objects represented in their own color.

The top servo performs the power stroke and rotates through 180 degrees (horizontal to horizontal), then the middle servo rotates in the same direction, before the tip returns to the original position; the middle servo then also returns to its original position. This action closely mimics the power stroke and recovery stroke (Figure 3). The similarity between the real ciliary beat and the beat of artificial cilia is demonstrated by comparing Figure 3 and Figure 8; the cardioid shape of the power and recovery strokes is evident. The mean force of the ciliary beat based on 12 repeats is (1.38 ± 0.29) N. A video of the beating action is available (Supplementary Video S1), triggered by moving a finger over the color sensor, the sensor detects a red object, then moved to the appropriate angle and performs a ciliary beat. The power and recovery strokes are clearly visible. Some examples of object manipulation can be seen in Supplementary Video S2 with different colored undersides, and the parcel is moved in different directions; three examples for each colored object are demonstrated in Figure 9, showing the different directions for red, green, and blue objects (with appropriate line coloring).

**Figure 10:**
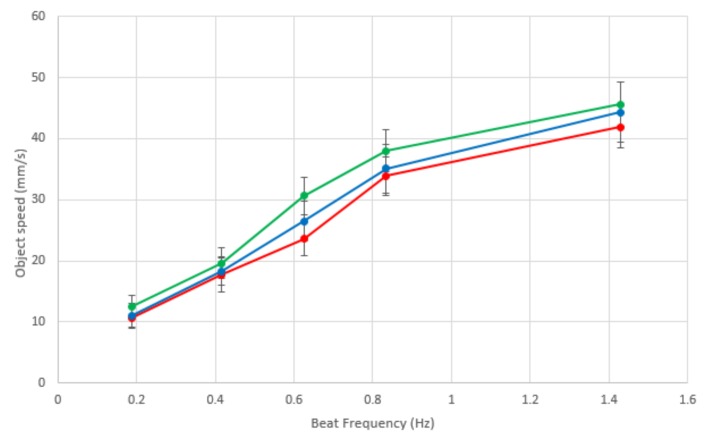
The effect of robotic cilia beat frequency on the speed of an object being moved across the surface. Each line is the same color as the object, illustrating red, blue and green objects.

Table 1 and Figure 10 show the proportional relationship between ciliary beat speed and object speed; increasing the frequency of ciliary beating increases the mean object speed across the surface.

### 3.1. Manipulation Based on Shape

**Figure 11:**
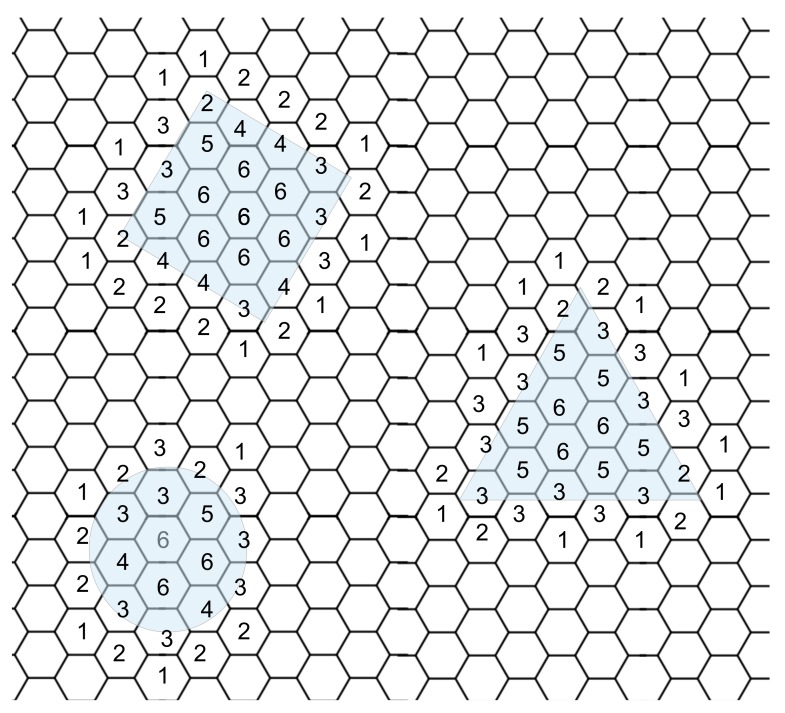
Cells nearby objects and under the objects display a number of neighbors covered by an object. The shapes are shown by the blue transparent boxes and the numbers indicate how many local neighbours are covered.

Attempts to identify corners to determine the shape of an object proved very difficult. Using specific neighbor classifiers meant that corners were misidentified and edges were incorrectly identified as corners. The variability of object orientation and placement over cells meant that even for the same corner, corners were not identified in some circumstances. Given the addition of the hexagonal tiling, a corner in one orientation could be identified, however in another orientation was missed. Since the identification of corners using this method was not satisfactory, even with significantly scaled neighbor communication complexity, the identification of shapes was insufficient to be able to reliably manipulate objects using their shape. Heat maps of neighbor activity (Figure 11) roughly approximated the shape above them; however, due to the hexagonal nature, some shapes were indistinguishable, such as the circle and square. Increasing the number of sides of polygons would lead to further inaccuracy of shape detection unless much larger shapes were tested to limit the effect of cell-to-shape resolution ratio; larger shapes would however mean the distance between corners would be much larger, requiring the sharing of several concentric neighbor states, increasing communication and geometrical complexity beyond that reasonably practical.

### 3.2. Edge Detection

**Figure 12:**
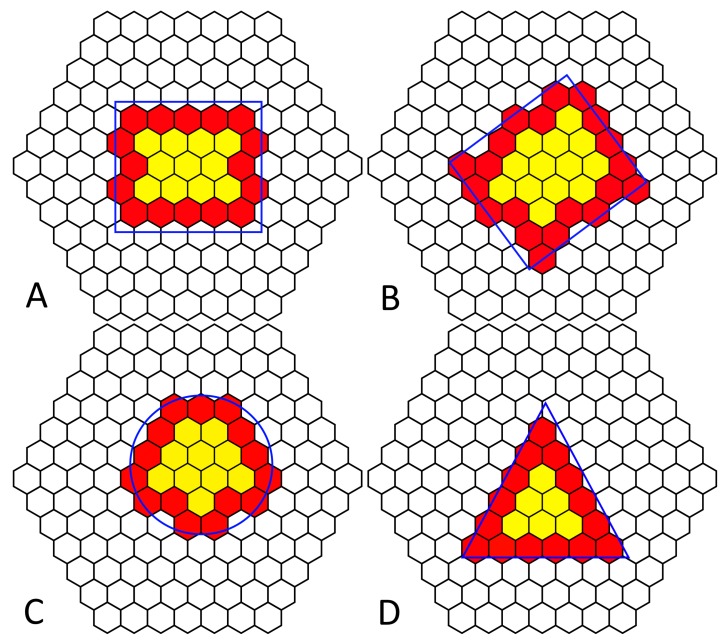
Edge detection of various simple shapes (blue outline) on a tiled hexagonal cilia platform (black). Clear hexagonal cilia are “uncovered”, red are “edge”, and yellow are “interior” cilia.

Several shapes were placed over the object in different orientations to test the edge detection accuracy. The output of the cells was given three categories: (1) uncovered, (2) edge, and (3) interior. If the cell had no item above it, it fell into the “uncovered” category; if a cell had an item above it and at least one neighbor that was uncovered, it formed an “edge”; while covered cells with no uncovered neighbors were part of the “interior” of the shape. Figure 12 demonstrates the outcome of placing different shapes of objects on the platform with cells that were “uncovered” white, while “edge” cells red and “interior” cells yellow. Edges of the cells were detected successfully, and no cells were inaccurately determined. The platform’s approximation of the shape of the object varied depending on placement on the platform and shape. While some cells are partially covered, their cell status is dependant on whether the object covered the RGB sensor. The accuracy of certain shapes such as the circle was the least accurate (Figure 12C), perhaps due to the placement of the circle on the board. Even in cases where the object’s shape was accurately detected (square), the hexagonal design of the boards meant it was impossible to get a straight line vertically, as the tiles are not aligned in a traditional square matrix or grid. Smaller objects were not accurately detected as the size of the object was too small compared to cells, meaning the resolution of the number of cells was too poor. Increasing the size of the object means that a more accurate shape estimate could be obtained, due to the higher resolution of cells to the surface area of the shape. The accuracy of each shape was determined by calculating the area of the cells that detected the object above and working out the error from the area of the shape. The circle error was 10.18%; square error was 13.3%; and triangle error was 5.79%.

Emergent behavior is observed when locally coordinated cells are governed by a simple set of rules; each cell has the same preprogramed behavioral response, and using knowledge of its neighboring cell states, rotation of the object occurs even though the cells themselves do not know the shape or size of the object.

## 4. Discussion

The actuation platform presented here has demonstrated differential manipulation of objects of different colors. Its *Paramecium*-like biomimetic features include decentralized control, parallel actuation, instantiation of metachronal waves and hexagonal arrangement. Although the organism the device is designed after cannot actively differentiate between colors and possesses only two orientations of beat direction (anteroposterior and posteroanterior), the device presented is nevertheless highly analogous to *P. caudatum* ciliary fields in their mode of beating and method of discrimination of the properties of adjacent materials. Sorting by color in this instance was accurate for all tests. The colors chosen were easy to distinguish for an RGB sensor; however, if the colors were closer together, or there were more categories of color, there may be instances where the colors would be incorrectly identified. As we have stated, this proof-of-concept parallel distributed sorting platform can be adapted for a practical application. Where color is irrelevant, sorting may be performed by a variety of other sensors. In a post or luggage sorting office, barcode scanners may be employed on each hexagon cell in order to coordinate behavior, or in a recycling environment, different sensors can be employed to distinguish between categories of recycling. The plug-and-play nature of the cells provides a wide range of possibilities; for example, one could have several regions within the platform that perform different tasks. The value of decentralized control is realized here; without the need for complex central processing unit (CPU) algorithms dictating actuation, often with the need for image processing and a vast range of parallel connectivity, we have demonstrated object manipulation using a hexagonal grid of sensor–actuator cells that coordinate behavior by fusing sensing with local neighbor states. This system demonstrates the flexibility of a simple system to behave in a complex manner, much like the emergent properties of swarms or multiagent cellular systems [10].

The accuracy of the platform’s estimation of the shape varies between 5.79% and 13.3%; however, the shape detection relies on having at least one outer layer of empty cells. The platform is able to detect multiple objects on the same surface, provided the surface is large enough and that there is at least one cell gap between the objects. If there is no gap, then the platform will see multiple objects as one large complex polygon. In situations where multiple objects are placed on the platform, then color could be used to determine where multiple objects appear as one. In some circumstances, edge detection could be flawed; where multiple, similar objects are next to each other or overlapping, they may appear to be the same object, whereas one object with multiple colors on it may appear as several smaller objects. The implementation of edge detection should therefore be application dependant. In order to accurately process edges, we use the assumption that there are no holes in the objects and that any concave edges are not smaller than the size of a cell, otherwise the edge may not be detected; however, this may not cause issues in every scenario, but it may lead to erroneous operation.

The control system for this device allows for local communication, therefore coordination of behavior. Coordination is rarely performed between neighboring cells or actuators; in most closed-loop cilia-like manipulation surfaces, control and coordination of the cilia actuators is performed by a separate computer, which controls each actuator in parallel. This device is a significant advancement from our previous generation of local-neighbor coordinated control [13] in that is has a stronger ciliary beat resemblance. It also can distinguish between different objects, discriminating by color, whereas the previous generation could only manipulate objects based on their presence. One limitation of the current generation of ciliary sorting platforms is that they currently do not handle multiple simultaneous objects traveling towards each other; this may not be a problem depending on the application, if objects are placed on the center of the platform and moved to specific locations around the perimeter, for example with mail sorting or airport luggage sorting situations. It would be ideal, however, to facilitate the simultaneous sorting of multiple objects traveling in different directions, requiring some complex or emergent collision avoidance algorithm. There is a clear positive relationship between ciliary beat frequency and object speed across the platform; it was not possible to beat faster than 1.4 Hz due to the limitations of the speed of the servos and the sequence of their motion to mimic the cyclical cilia beat pattern. The discrepancy between speeds when transporting different colored objects is due to the control, the slight difference in time that the base servo takes to change its position to manipulate an object in a different direction. It could be possible to coordinate multiple objects at different speeds to avoid collisions. Impairment to ciliary beating such as in patients with Primary ciliary dyskinesia leads to poor mucociliary clearance; our results highlight the issues with slower beating speed and confirm the importance of healthy cilia.

While this work has maintained the directive of biomimicry, as with any project inspired by nature, unless the hardware equivalent is identical to the natural counterpart, then there has to be a trade-off between similarity and engineering efficiency. In the field of biomimicry, the design criteria are often to produce innovative solutions to problems, using nature’s engineering precedent. The similar field of bioinspiration has many mutual principles and ideals: that of using nature as inspiration for engineering tasks. There is significant overlap; however, the field of bioinspiration suggests less of a cloned design; rather, one can cherry-pick the best biological features from nature and apply them to a wider range of tasks. *Paramecium* swim through water, sorting and sifting food particles while avoiding predation; the cilia facilitate these actions and thus are the biological source of inspiration. We have developed a bioinspired technology that is capable of sorting and transporting objects across its surface; the mimicry in this is the action of the ciliary beat, albeit several orders of magnitude larger. We are not attempting to provide aquatic locomotion or sorting of aqueous particles, so the physics of the water vortexes created differ from with application. The interesting feature of ciliary beating is the asymmetrical reciprocity of the tip, providing the microorganism with motility and an elegant actuator in this application. It is this characteristic that we have mimicked, through careful reproduction of the biological structure and function of *P. caudatum* cilia, as defined in Section 2.1.

## Figures and Tables

**Table 1:** Beat frequency effect on object speed.

Beat Frequency	Blue	Red	Green
(Hz)	(mm/s)		
0.2	11.7	10.7	12.5
0.4	18.3	17.7	19.6
0.6	26.5	23.6	30.6
0.8	35.1	33.9	38.0
1.4	44.3	41.9	45.6

## Supplementary Materials

The following are available at http://www.mdpi.com/2313-7673/3/2/5/s1, Video S1: Robotic ciliary beating, Video S2: Cilia sorting platform. All the files required to reproduce and reprogram the cilia are available on GitHub: https://github.com/UnconventionalComputingUWE/Paramecium.
